# Is Intensity Inhomogeneity Correction Useful for Classification of Breast Cancer in Sonograms Using Deep Neural Network?

**DOI:** 10.1155/2018/8413403

**Published:** 2018-12-04

**Authors:** Chia-Yen Lee, Guan-Lin Chen, Zhong-Xuan Zhang, Yi-Hong Chou, Chih-Chung Hsu

**Affiliations:** ^1^Department of Electrical Engineering, National United University, Miaoli, Taiwan; ^2^Department of Radiology, Taipei Veterans General Hospital and National Yang Ming University, Taipei, Taiwan; ^3^Department of Management Information Systems, National Pingtung University of Science and Technology, Neipu, Taiwan

## Abstract

The sonogram is currently an effective cancer screening and diagnosis way due to the convenience and harmlessness in humans. Traditionally, lesion boundary segmentation is first adopted and then classification is conducted, to reach the judgment of benign or malignant tumor. In addition, sonograms often contain much speckle noise and intensity inhomogeneity. This study proposes a novel benign or malignant tumor classification system, which comprises intensity inhomogeneity correction and stacked denoising autoencoder (SDAE), and it is suitable for small-size dataset. A classifier is established by extracting features in the multilayer training of SDAE; automatic analysis of imaging features by the deep learning algorithm is applied on image classification, thus allowing the system to have high efficiency and robust distinguishing. In this study, two kinds of dataset (private data and public data) are used for deep learning models training. For each dataset, two groups of test images are compared: the original images and the images after intensity inhomogeneity correction, respectively. The results show that when deep learning algorithm is applied on the sonograms after intensity inhomogeneity correction, there is a significant increase of the tumor distinguishing accuracy. This study demonstrated that it is important to use preprocessing to highlight the image features and further give these features for deep learning models. In this way, the classification accuracy will be better to just use the original images for deep learning.

## 1. Introduction

In recent years, with dietary habit and the change of lifestyle, a quick rise has been seen in female's breast cancer. In the developed and developing countries, breast cancer has, respectively, become the first and second causes of women's cancer death. According to the article published in International Journal of Cancer, 1993, one in every 8 women in European and American countries has breast cancer [1]. American Cancer Society pointed out as early as in 1999 that breast cancer is the first cause of women's death in the developed countries [2]. In the various breast cancer diagnosis procedures, ultrasound diagnosis is regarded as a kind of “highly accepted” tool because of low price, convenience, universality, and nonradiation. Ultrasound has become a necessary diagnosis tool in all medical centers and a good tool for doctors to initially diagnose the breast cancer clinically.

However, ultrasonic images often contain lots of speckles, noises, and textures so that it is unable to clearly discriminate the detail changes of tissues, e.g., the tumor size and boundary. Therefore, many studies in the past have put forward ultrasound tumor segmentation algorithm, including the well-known deformation model [3, 4], clustering [5], overzero [6], thresholding [7], watershed method [8], and level set method [9, 10]. The above methods can be roughly divided into two categories, respectively, based on pixel or region information [11]. However, in order to make the segmentation results well [12–14], many experiment steps and parameter adjustments are involved. It may have been affected by the developer's subjective ideas and habits so that these results may be not sufficiently objective, affecting the accuracy in doctor's judgment with naked eyes, and it is likely to cause misdiagnosis. Therefore, the traditional computer-aided diagnosis tool is limited.

Recently, deep learning has been widely used in various applications such as facial recognition [15, 16], object detection, and car identification. In medical imaging, deep learning still offers excellent performance on different fields [17, 18]. With the development of deep learning, the system architecture of computer-aided diagnosis has been changed, and the advantages unavailable in traditional computer-aided diagnosis system have been added. In the deep learning method, it is only needed to specify the training data, and then, the features in the images can be automatically extracted, and more abstract feature descriptions can be extracted according to the nerve cell depth of each layer, including the features from point to edge, contour, and even a higher level so as to gradually reduce the nerve cells at the same time of improving the features. In the deep learning method, selection and extraction of features, as well as data classification, are established under the same structure, which has a higher accuracy than traditional feature extraction.

To address the problem of the breast sonogram detection, there are several approaches that can be used such as a convolutional neural network (CNN) [19–21], deep autoencoder (DAE), or stack denoised autoencoder (SDAE). Zhou et al. demonstrated the importance of feature extraction and selection for tumor classification and confirmed that the classification effect based on the Shearlet-based texture feature was better than those of the other four feature extraction methods by AdaBoost and SVM. In the classification of 200 benign and malignant tumors, the accuracy is up to 90%, indicating the effectiveness of using machine learning for breast tumor classification [22]. In 2017, Moon added adaptive filtering to the CAD system (A-CAD) to emphasize the characteristics of tumor size. CAD was more robust when classifying tumors larger than or equal to 1 cm. The results showed that the accuracy increased from 73.1% to 81.4% after adding the adaptive filter. It is suggested that the tumor classification effect is better after using adaptive filtering [23]. Due to the speckle noise of ultrasonic images, Raha used four kinds of filters (median, high boost, Sobel, and average filter) for image preprocessing, and watershed was used for segmentation. Finally, the k-nearest neighbors algorithm was performed for the classification of benign and malignant tumors with a high accuracy of 96.4% [24]. Abdel-Nasser extracted a high-resolution (HR) image from a set of low-resolution (LR) images using super-resolution to overcome the speckle noise and artifacts problems. The 31 benign and 28 malignant tumors were divided into training and test data by leave-one-out cross validation (LOOCV) and classified by random forests [25]. The classification results with high accuracy were obtained. It was also emphasized that if the texture features were able to accurately represent the tumor information, it is very important to perform preprocessing. Cheng et al. conducted research on deep learning-based CAD. To avoid using inaccurate image processing results to achieve tumor classification, the team used SDAE for US breast cancer, pulmonary nodules in CT scans classification. Relying on the advantages of SDAE well equipped with the automatic feature exploration mechanism and noise tolerance advantage, the classification result can reach 82.4% accuracy. Cheng et al. reported that the research is the first CAD study based on deep learning, which can further study other image features for more accurate classification results [26]. Overall, it is important to do image preprocessing to highlight the image features and further give these features for deep learning models.

In general, CNN is an end-to-end architecture and suitable for various image classification tasks. However, it is relatively hard to find the useful and meaningful features in a constrained-scale training set. In breast sonogram classification task, the training set is usually smaller than the size of the general training images. For example, the number of training images in ILSVRC12 [19] is 1.2 million, while that of our training set is 170. Instead, the autoencoder families deal with feature extraction task as a data reconstruction task. By minimizing the reconstruction error, the primary feature of the training images should be detectable. In this study, therefore, we tend to adopt the autoencoder network to extract the useful features and follow by concatenating a classifier to distinguish the type of breast sonogram. SDAE [27] is one of the state-of-the-art autoencoder architectures. Xing et al. adopted SDAE for the extraction of tumor features and classification of benign and malignant tumors [27]. SDAE is about to find out many typical patterns in the input training data, to deal with the high change problem of tumor margin, and moreover, it is provided with multilayer training automatic feature extraction and noise reduction ability so that the problems likely to occur under traditional methods may be avoided.

The accuracy of classifier is highly related to the quality of test images. As for ultrasonic image, due to the imaging principle and the properties of object to be tested, the research scholars are often encountered with two major problems, much noise and intensity inhomogeneity. Noise-resistant function has been added to SDAE when it is designed, to reduce the noise effect. In terms of intensity inhomogeneity, studies have found that after the preprocess of intensity inhomogeneity correction [28–31] of medical images, the imaging quality is effectively improved so as to reach a better image processing result. There are several intensity inhomogeneity correction methods. For example, the filter method [32, 33] is to take intensity inhomogeneity as low frequency signal and eliminate the intensity inhomogeneity with the filter method, but such a method has the elimination risk of important low-frequency information; the surface fitting method [34] is generally used to establish a curve with polynomial or spline, and the characteristics in the image are fit on such a curve to serve as image intensity inhomogeneity; the histogram method is to take the observed image as the convolution of original image and intensity inhomogeneity, and the original image can be obtained only through deconvolution; the image segmentation method [35, 36] is able to get the segmentation result and estimated intensity inhomogeneity; or the method combining with many concepts above is adopted [37]. In this study, intensity inhomogeneity correction is conducted before the input of ultrasonic testing images. Then, the corrected images are input into the classifier system established on the basis of SDAE, hoping to overcome the effect of intensity inhomogeneity in ultrasonic imaging for improving the system resolution and quality.

Main aims of the paper are as follows:

- A classification algorithm in breast sonogram without subjective influences is proposed
- Up to 85% accuracy can be obtained when deep learning was applied to the intensity inhomogeneity-corrected image
- The study overcomes drawbacks of sonogram and has reliable discrimination ability
- It has the potential to obtain the better classification results after image preprocessing

## 2. Materials and Methods

### 2.1. Input Data

This study was approved by the Institutional Review Boards of the Taipei Veterans General Hospital. All the experimental methods were carried out in accordance with the approved guidelines. In this study, we used two kinds of dataset. One is the data collected by our team (a database of 96 malignant and 74 benign images) and the other is the public dataset on the website, Rodrigues, Paulo Sergio (2017), “Breast Ultrasound Image,” Mendeley Data, v1 (a database of 150 malignant and 100 benign images) [38].

### 2.2. SDAE

Artificial neural network is a kind of machine learning concept to simulate the learning of human's brain. As for each nerve cell, the nerve cell signals connected to it will influence the output of such nerve cell. The transmission of nerve signals is to add the received signals and then conduct nonlinear transformation, to get new output. The mutual connection in neural network decides the calculation method to make the actual output approach the expected output as much as possible through neural network adjustment.

The training of one neural network is to limit the output value to be equal to the input value, and this indicates that the output layer has the same quantity of nerve elements as the input layer, and the error between the two layers is used to adjust the weight of each layer. The training of autoencoder (AE) is unsupervised, so no label information is needed. Denoising autoencoder (DAE) is improved on the basis of AE. It is assumed that the input data include noise, so DAE is suitable to learn the features from the data including noise. SDAE is stacked by DAE, to obtain higher level features. The network training of SDAE is layer-wise, for it is of independent training between each DAE. As for the SDAE network after training, the decoding layer is eliminated, while the encoding layer generating features is reserved. To classify the data, logistic regression (LR) layer is added to be taken as output layer, and LR is of supervised type, adding the expected output volume label information through backpropagation algorithm, and based on the error between actual output and expected output, the network weight between layers is fine-tuned. Therefore, the feature learning of system is the result of combination between SDAE pretraining and LR adjustment.

**Figure 1:**
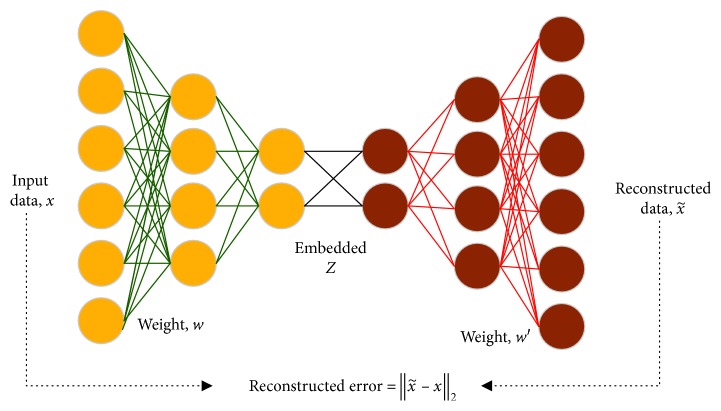
The flowchart of the reconstructed error of the DAE used in this paper.

It is supposed that the input data are *x* and DAE antinoise method is used to firstly set some input as 0 or add Gaussian noise to generate $\tilde{x}$ as shown in Figure 1. The way of thinking is to make the input not perfect in the very beginning so as to get better features after training. Therefore, a good result still can be achieved after the input is polluted. DAE encode is obtained according to one nonlinear transformation equation:

(1) $$\begin{array}{c} Z=f_{\theta }\left(w\tilde{x}+b\right), \end{array}$$

where *Z* is the output of the hidden layer, called as feature description, *w* is the weight of input and hidden layers, *b* is the offset, and *f*_*θ*_ is the activation function of the hidden layer. DAE decoding and reconstruction are realized through the mapping function:

(2) $$\begin{array}{c} x^{\prime }=g_{\theta^{\prime }}\left(w^{\prime }y+b^{\prime }\right), \end{array}$$

where *x*′ denotes the transpose operation, *x* the output of DAE (the reconstruction), and w the corresponding weight. DAE may generate a depth network with many hidden layers through stacking. A standard SDAE structure includes two encoding layers and two decoding layers. In the encoding stage, the output of the first encoding layer is taken as the input of the second encoding layer. It is supposed that there are *L* hidden layers in the encoding stage, and we can obtain the activation function of number *k* encoding layer as follows:

(3) $$\begin{array}{c} Z^{\left(k+1\right)}=f_{\theta }\left(w^{k+1}Z^{k}+b^{k+1}\right),\;k=0,\ldots ,L-1, \end{array}$$

where *Z* ^0^ is the original input and *Z* ^*L*^ is the feature description of the highest layer. The decoding stage is the same as the encoding stage. The output of the first decoding layer is taken as the input of the second decoding layer, and in this way, the activation function of number *k* encoding layer can be obtained as follows:

(4) $$\begin{array}{c} x^{\prime \left(k+1\right)}=g_{\theta^{\prime }}\left(w^{\left(L-k\right)T}x^{\prime k}+b^{\prime \left(k+1\right)}\right),\;k=0,\ldots ,L-1, \end{array}$$

where *x* ^′0^ is the output of the last layer in the encoding stage and *x* ^′*L*^ is the reconstruction of *x*. The weight of SDAE after pretraining will be the initial weight of adjustment and classification stages. Once the import and discriminative features have learned by SDAE, the next step is to find a suitable classifier to correctly detect the type of the extracted feature of the input image. Since there are many different classifiers such as linear classifier, perceptron, SVM, random forest, and so on, it is necessary to select one of them for the best performance purpose. Most of them are different from SDAE, while the perceptron is also based on the neural network. Inspired by perceptron learning, we concatenate a sigmoid layer to SDAE and formulate it as a cross-entropy loss function to keep it as an end-to-end architecture.

In the LR stage, the expected output is added, and the weight after pretraining is fine-tuned through the supervised learning method, and the activation function of LR layer is *S* (sigmoid):

(5) $$\begin{array}{c} S\left(t\right)=\frac{1}{1+\exp\left(-t\right)}, \end{array}$$

where *t* is the output *Z* ^*L*^ of the final encoding layer and also the depth feature of pretraining of the SDAE method, and the output of *S* is the classification result, with the value ranging from 0 to 1.

The backpropagation algorithm is one kind of gradient descent methods. After the gradient direction is worked out, the weight of the classifier system will advance towards the direction with the quickest gradient descent. In addition, it is a kind of the greedy method, for it always advances towards the steepest direction, seeking for the biggest decent extent.

To realize the slight adjustment of weight, the error calculation method shall be defined. Different error calculation methods have different weight upgrading rules. Here, the common square error will be adopted:

(6) $$\begin{array}{c} E=\frac{1}{2}\underset{d\in D}{\sum }{\left(t-y\right)}^{2}, \end{array}$$

where *D* indicates all input data, *d* indicates one of the input data, *t* is the expected output, and *y* is the actual output.

The target is to find out a set of weights, to realize the smallest calculation error:

(7) $$\begin{array}{c} w=w+\Delta w,\\ \Delta w=-\alpha \frac{\partial E}{\partial w}, \end{array}$$

where *α* indicates the learning rate. The weight upgraded mode is the batch mode. After all inputs are executed, the connecting weight will be changed.

### 2.3. Intensity Inhomogeneity Correction

The algorithm was composed of two parts, constrained fuzzy cell-based bipartition and the intensity inhomogeneity modelling [39]. When correcting the intensity inhomogeneity, the polynomial surface fitting method is adopted to estimate the image intensity inhomogeneity. Curve fitting is a kind of concept to represent the existing data through the mathematical method. Fitting is to obtain the discrete information through sampling and testing in engineering. Based on the data obtained in this way, it is hoped to obtain a continuous function (polynomial) or the gathering (spline) of many discrete equations to be identical to the data. Surface fitting is the popularization of curve fitting. It is supposed that the region of interest (ROI) image is composed of foreground (*F*) and background (*B*), and each area is regarded as the homogeneous area.

The observed gray level of image can be expressed as follows:

(8) $$\begin{array}{c} O_{i}=\mu_{\psi }+P_{i}+n_{i}, \end{array}$$

where *O*_*i*_ is the gray level of number *i* pixel, *μ*_*ψ*_ is the average value of area where the pixel is located, *ψε*{*F*, *B*  } represents the area where the pixel is located, and the latter two items indicate intensity inhomogeneity and noise. Here, intensity inhomogeneity is supposed as the normal distribution of spatial changes, which is composed of a fixed variance and the average value *P* changing with the space, and it is represented with polynomial surface form. *P*_*i*_ represents the value of polynomial in number *i* pixel, that is, the average value of intensity inhomogeneity of number *i* pixel in the image, and *n*_*i*_ is the composition of change and noise in the polynomial surface indicating intensity inhomogeneity.

Adopt the least square method to minimize the cost function and estimate the polynomial surface as follows:

(9) $$\begin{array}{c} \varepsilon^{2}=\frac{1}{N}\underset{\forall i}{\sum }{\left(O_{i}-\mu_{\psi }-P_{i}\right)}^{2}. \end{array}$$

When the pixel is in the foreground area, *μ*_*ψ*_=*μ*_*F*_, and when the pixel is in the background area, *μ*_*ψ*_=*μ*_*B*_. *P*_*i*_=*P*(*x*_*i*_, *y*_*i*_), in which *P* is *N* time polynomial. After the polynomial surface is obtained, the image intensity will be adjusted based on results, to complete the image correction.

The image training and classification steps are shown as follows:

1. Input training image and initialize the neural network weight by use of SDAE
2. Remove decoding part and add LR structure, to establish SDAE-LR system
3. Add expected input volume label and slightly adjust the network weight, to complete the classifier

**Figure 2:**
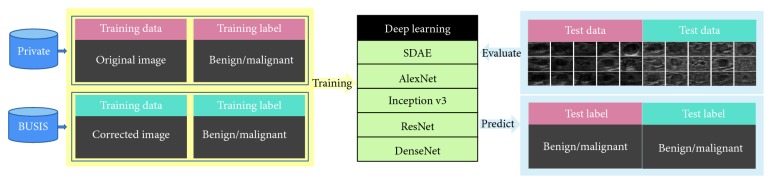
The flowchart for deep learning.

We use the original images of the two different databases, i.e., private dataset and BUSIS dataset, and conduct intensity inhomogeneity correction for the original images. Therefore, each database will generate two groups of images (the original image and the corrected image) and their labels as training data. These materials are trained in the deep learning model and then performed testing to predict the benign/malignant lesions in the image. In this study, we use five different models to compare the results.  Figure 2 shows the flowchart for deep learning.

## 3. Results and Discussion

**Figure 3:**
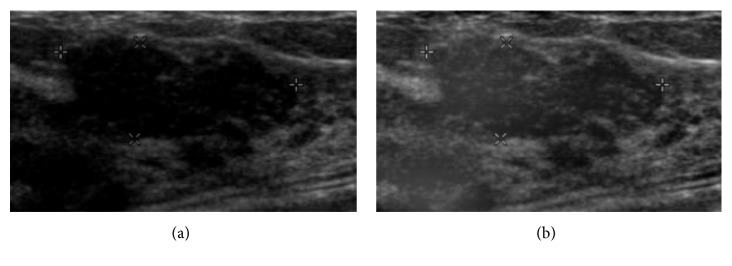
Malignant: (a) original image and (b) image after correction.

Intensity inhomogeneity often causes unclear contour of tumors in sonogram so that it is uneasy to judge the type of tumors clinically. The correction method is proposed for improving image quality and increasing the accuracy in classification.  Figure 3 shows the comparison images of one malignant tumor with/without intensity inhomogeneity. Before correction, it is uneasy to find the whole edge of the tumor. There is a missing boundary part at the left bottom of the tumor so that a wrong judgment may be caused. However, after correction, the missing part can be clearly seen, so it could be correctly judged as a malignant tumor.

**Figure 4:**
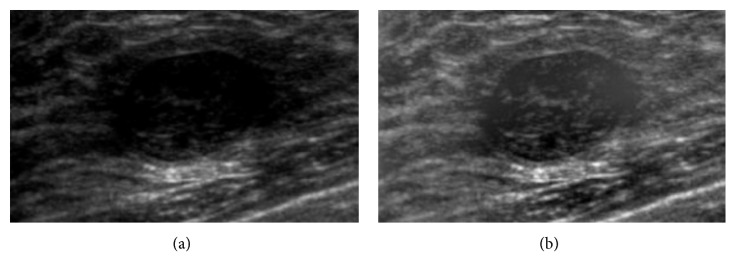
Benign: (a) original image and (b) image after correction.

Certainly, intensity inhomogeneity correction also works well in benign tumor images.  Figure 4 shows the comparison images of benign tumor with/without intensity inhomogeneity. The original image with intensity inhomogeneity has unclear boundary at the right and left of the tumor that would cause wrong classification result. After correction, the unclear boundary part can be obviously distinguished so as to classify the type of the tumor correctly.

In this study, we used two kinds of dataset. One is the data collected by our team, named private dataset (96 malignant and 74 benign images), and the other is the public dataset on the website, named BUSIS dataset (150 malignant and 100 benign images). In the private dataset, 143 images and 27 images are selected randomly as training data and test data, respectively. In the BUSIS dataset, 210 images and 40 images are selected randomly as training data and test data, respectively.

**Figure 5:**
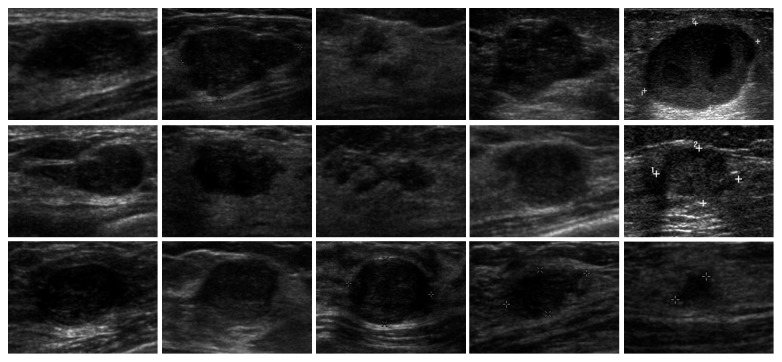
The examples of original test images in the private dataset.

**Figure 6 fig6:**
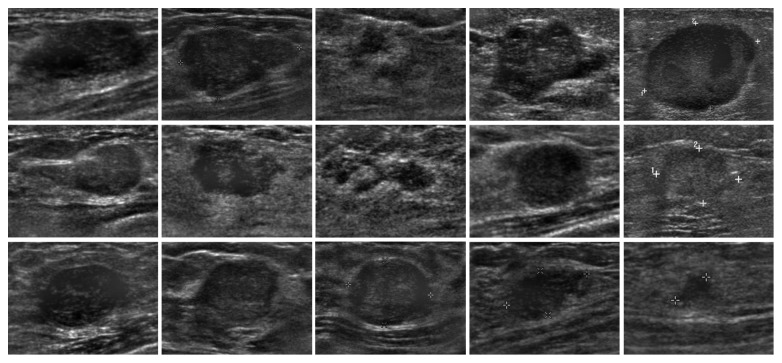
The examples of test images after correction in the private dataset.

**Figure 7 fig7:**
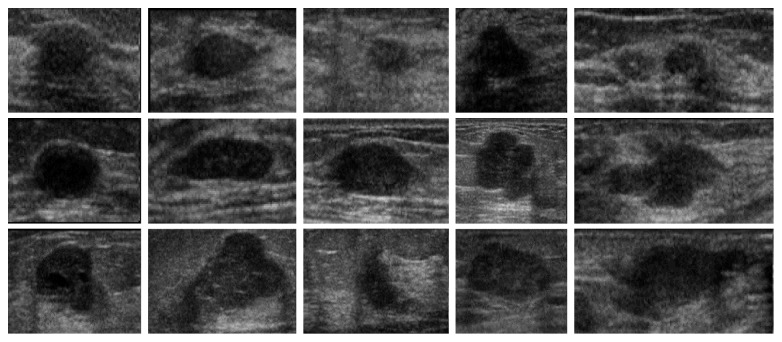
The examples of original test images in the BUSIS dataset.

**Figure 8:**
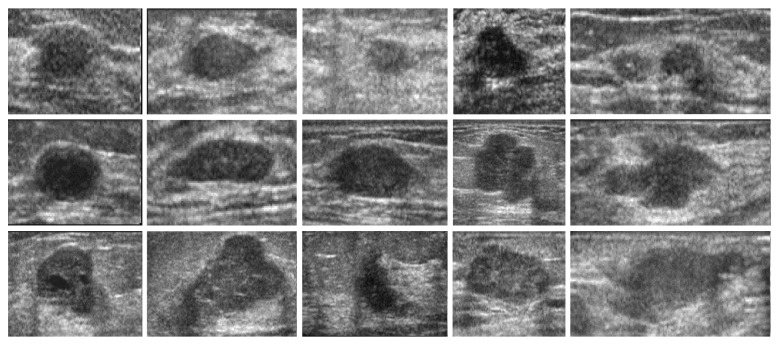
The examples of test images after correction in the BUSIS dataset.

First, we feed ultrasound tumor ROI images into SDAE network sized of 28 × 28. In the private dataset, 143 images and 27 images are selected randomly as training data and test data, respectively. In the BUSIS dataset, 210 images and 40 images are selected randomly as training data and test data, respectively. Second, a backpropagation algorithm is used on SDAE to learn the weights and the feature representation. Third, the images are labeled manually and conducted the second supervised training to classify the malignant/benign tumor images. The experiments of benign/malignant classification are grouped into two groups: (1) original images and (2) contrast-enhanced images by intensity inhomogeneity correction, which are shown in Figures 5 [Fig fig6] [Fig fig7]–8, respectively.

For the private dataset, the classification accuracy values of testing results of two groups of images are, respectively, 63% and 82%. For the BUSIS dataset, the classification accuracy values of testing results of two groups of images are, respectively, 75% and 83%. The results indicate the improvement of image quality (contrast-enhanced images) actually achieve better classification performance. The results also show that because the image complexity in the private database is higher, the accuracy increases noticeably after intensity inhomogeneity correction.

As we stated previously, CNN may fail when the number of the training images is relatively small. To verify this, we also conduct an experiment of CNN for detecting benign and malignant tumors. The labeled training images are directly fed into AlexNet [19] to learn the prediction results (i.e., benign or malignant tumors). Similarly, the backpropagation algorithm is used to train the weights of AlexNet. Finally, the classification accuracy values of testing results of two groups of private dataset images are 44% and 37%, while the training accuracy values are 99.8% and 99.4%, respectively. Apparently, such CNN architecture is not suitable for such small-scale training images, leading to a serious overfitting problem. It also verified that the proposed SDAE is a proper choice for analyzing malignant/benign tumor images classification. In addition, three models (Inception v3 [40], ResNet [41], and DenseNet [42]) are also used for comparison.  Table 1 shows the detection results of the original images (without correction) and the corrected images (with correction) of the private database, in which the displayed value means with/without intensity inhomogeneity correction, respectively.  Table 2 shows the results of the BUSIS dataset. To sum up, because the complexity of the images in the BUSIS database is lower, the classification accuracy is higher than that of the private database before or after intensity inhomogeneity correction.

It is clear that the DenseNet achieves the best performance. However, it is also shown that the performance of the different CNN methods cannot achieve good performance, compared with SDAE approach. Although the number of the parameters of AlexNet is relatively low, the total number of parameters is still greatly large than that of SDAE. By fair comparison, we have chosen Resnet with 54 layers and Inception v3 with 34 layers to learn the classifiers with the default settings suggested by their approach. However, such lot parameters in these two networks lead to serious overfitting problem and bad test performance. On the contrary, the most advanced network architecture, DenseNet, achieves good performance among four CNN methods; it still fails to obtain promising performance at all.

In [43], this study discriminates benign cysts from malignant masses in breast ultrasound by transferred deep neural network, Inception V3, with an accuracy of 89.44%. However, this study uses a total of 2058 breast ultrasound masses, comprising 1370 benign and 688 malignant lesions, so better results can be obtained. In [44], Han et al. use GoogLeNet with preprocessing for supporting the classification of breast lesions in ultrasound images. The networks showed an accuracy of about 90%. This study uses large data, 7408 images (4254 benign and 3154 malignant lesions), to get a good classification result. Apparently, CNN architecture is not suitable for such small-scale training images, leading to a serious overfitting problem. It also verified that the proposed SDAE is a proper choice for analyzing malignant/benign tumor images classification. In addition, Han et al. also proposed that the preprocessing can get better classification results.

## 4. Conclusion

This study reports that the proposed algorithm overcomes the problem of intensity inhomogeneity in the sonogram, and in combination with the deep learning method for tumor classification. This study compared five deep learning models, and SDAE achieved the best identification accuracy. Since this is a small amount of data, SDAE is a suitable choice for analyzing the small dataset.

For the original image group and the corrected image group in the private database, the accuracy values of identifying the benign and malignant tumors are 63% and 82%, respectively. For the same group in the public database, the accuracy values are 75% and 83%, respectively. The results refer that increasing the image quality will help improve the accuracy of classification. The image complexity in the private database is higher. From the results, it can be inferred that for images with high complexity, the classification accuracy increases noticeably after image preprocessing.

This work is different from the traditional algorithm to classify the type of lesions, i.e., benign or malignant in the sonogram. In the traditional way, the tumor contours must be delineated first by the segmentation technique before they can be classified. However, many segmentation algorithms need to adjust many parameters or depend on the developer's subjective ideas. In this way, the result of the segmentation in turn affects the classification result. The proposed method reduced the impact of segmentation processing steps to lead an objective classification result via deep learning. Moreover, this paper combines an intensity inhomogeneity correction method to make the classification result better. This way is advantageous, and it has the potential to obtain the better classification results after image preprocessing.

## Figures and Tables

**Table 1:** Classification results of the private database (the displayed value: original/corrected).

	SDAE	AlexNet	Inception v3	ResNet	DenseNet
TP	8/11	5/6	6/7	7/7	6/6
TN	9/11	7/4	6/5	6/7	8/7
FP	4/2	7/10	8/9	8/7	6/7
FN	6/3	8/7	7/6	6/6	7/7
Precision	0.67/0.85	0.42/0.38	0.43/0.44	0.5/0.5	0.5/0.46
Recall	0.57/0.79	0.38/0.46	0.46/0.54	0.46/0.54	0.46/0.46
Specificity	0.69/0.85	0.5/0.28	0.43/0.36	0.57/0.5	0.57/0.5
Accuracy	0.63/0.82	0.44/0.37	0.44/0.44	0.51/0.52	0.52/0.48
*F*-measure	0.62/0.85	0.4/0.41	0.44/0.48	0.48/0.52	0.48/0.46

**Table 2:** Classification results of the BUSIS database (the displayed value: original/corrected).

	SDAE	AlexNet	Inception v3	ResNet	DenseNet
TP	17/18	10/16	13/15	16/16	15/17
TN	13/15	12/14	14/16	13/10	14/13
FP	7/5	8/6	6/4	7/10	6/7
FN	3/2	10/4	7/5	4/4	5/3
Precision	0.71/0/78	0.56/0.73	0.68/0.79	0.69/0.61	0.71/0.71
Recall	0.85/0.9	0.5/0.8	0.65/0.75	0.8/0.8	0.75/0.85
Specificity	0.65/0.75	0.6/0.7	0.7/0.8	0.65/0.5	0.7/0.65
Accuracy	0.75/0.83	0.55/0.75	0.68/0.78	0.73/0.65	0.73/0.75
*F*-measure	0.77/0.83	0.53/0.76	0.67/0.77	0.74/0.70	0.73/0.77

## Data Availability

The data used to support the findings of this study are available from the corresponding author upon request.

## Abbreviations

AE: Autoencoder
DAE: Denoising autoencoder
SDAE: Stack denoising autoencoder
LR: Logistic regression
ROI: Region of interest.

## References

[1] Parkin D. M., Pisani P., Ferlay J. (1993). Estimates of the worldwide incidence of eighteen major cancers in 1985. *International Journal of Cancer*.

[2] Society A. C. (1999). Breast cancer facts & figures 1997-1998. https://www.nrc.gov/docs/ml0716/ml071640135.pdf.

[3] Chalana V., Linker D. T., Haynor D. R., Kim Y. (1996). A multiple active contour model for cardiac boundary detection on echocardiographic sequences. *IEEE Transactions on Medical Imaging*.

[4] Chen D. R., Chang R. F., Wu W. J., Moon W. K., Wu W. L. (2003). 3-D breast ultrasound segmentation using active contour model. *Ultrasound in Medicine & Biology*.

[5] Cigale B., Zazula D. (2004). Segmentation of ovarian ultrasound images using cellular neural networks. *International Journal of Pattern Recognition and Artificial Intelligence*.

[6] Clark J. J. (1989). Authenticating edges produced by zero-crossing algorithms. *IEEE Transactions on Pattern Analysis and Machine Intelligence*.

[7] Drew M. S., Wei J., Li Z. N. (1999). Illumination-invariant image retrieval and video segmentation. *Pattern Recognition*.

[8] Hill P. R., Canagarajah C. N., Bull D. R. Texture gradient based watershed segmentation.

[9] Lee S. H., Seo J. K. (2006). Level set-based bimodal segmentation with stationary global minimum. *IEEE Transactions on Image Processing*.

[10] Brox T., Weickert J. (2005). Level set segmentation with multiple regions. *IEEE Transactions on Image Processing*.

[11] Haddon J. F., Boyce J. F. (1990). Image segmentation by unifying region and boundary information. *IEEE Transactions on Pattern Analysis and Machine Intelligence*.

[12] Joo S., Yang Y. S., Moon W. K., Kim H. C. (2004). Computer-aided diagnosis of solid breast nodules: use of an artificial neural network based on multiple sonographic features. *IEEE Transactions on Medical Imaging*.

[13] Cheng J. Z., Chou Y. H., Huang C. S. (2010). ACCOMP: augmented cell competition algorithm for breast lesion demarcation in sonography. *Medical Physics*.

[14] Chang R. F., Wu W. J., Moon W. K., Chen D. R. (2005). Automatic ultrasound segmentation and morphology based diagnosis of solid breast tumors. *Breast Cancer Research and Treatment*.

[15] Le Q. V. Building high-level features using large scale unsupervised learning.

[16] Huang G. B., Lee H., Learned-Miller E. Learning hierarchical representations for face verification with convolutional deep belief networks.

[17] Shin H. C., Orton M. R., Collins D. J., Doran S. J., Leach M. O. (2012). Stacked autoencoders for unsupervised feature learning and multiple organ detection in a pilot study using 4D patient data. *IEEE Transactions on Pattern Analysis and Machine Intelligence*.

[18] Xu Y., Mo T., Feng Q. Deep learning of feature representation with multiple instance learning for medical image analysis.

[19] Krizhevsky A., Sutskever I., Hinton G. E. (2012). ImageNet classification with deep convolutional neural networks. *Advances in Neural Information Processing Systems*.

[20] Yap M. H., Pons G., Martí J. (2017). Automated breast ultrasound lesions detection using convolutional neural networks. *IEEE Journal of Biomedical and Health Informatics*.

[21] Xie X., Shi F., Niu J., Tang X. Breast ultrasound image classification and segmentation using convolutional neural networks.

[22] Zhou S., Shi J., Zhu J., Cai Y., Wang R. (2013). Shearlet-based texture feature extraction for classification of breast tumor in ultrasound image. *Biomedical Signal Processing and Control*.

[23] Moon W. K., Chen I. L., Chang J. M., Shin S. U., Lo C. M., Chang R. F. (2017). The adaptive computer-aided diagnosis system based on tumor sizes for the classification of breast tumors detected at screening ultrasound. *Ultrasonics*.

[24] Raha P., Menon R. V., Chakrabarti I. Fully automated computer aided diagnosis system for classification of breast mass from ultrasound images.

[25] Abdel-Nasser M., Melendez J., Moreno A., Omer O. A., Puig D. (2017). Breast tumor classification in ultrasound images using texture analysis and super-resolution methods. *Engineering Applications of Artificial Intelligence*.

[26] Cheng J. Z., Ni D., Chou Y. H. (2016). Computer-aided diagnosis with deep learning architecture: applications to breast lesions in US images and pulmonary nodules in CT scans. *Scientific Reports*.

[27] Xing C., Ma L., Yang X. (2016). Stacked denoise autoencoder based feature extraction and classification for hyperspectral images. *Journal of Sensors*.

[28] Dawant B. M., Zijdenbos A. P., Margolin R. A. (1993). Correction of intensity variations in MR images for computer-aided tissue classification. *IEEE Transactions on Medical Imaging*.

[29] Vemuri P., Kholmovski E. G., Parker D. L., Chapman B. E. Coil sensitivity estimation for optimal SNR reconstruction and intensity inhomogeneity correction in phased array MR imaging.

[30] Gerig G., Prastawa M., Lin W., Gilmore J. Assessing early brain development in neonates by segmentation of high-resolution 3T MRI.

[31] Ashburner J., Friston K. J. (2005). Unified segmentation. *NeuroImage*.

[32] Ardizzone E., Pirrone R., Gambino O. Exponential entropy driven HUM on knee MR images.

[33] Sled J. G., Zijdenbos A. P., Evans A. C. (1998). A nonparametric method for automatic correction of intensity nonuniformity in MRI data. *IEEE Transactions on Medical Imaging*.

[34] Hernández J. A., Mora M. L., Schiavi E., Toharia P. RF inhomogeneity correction algorithm in magnetic resonance imaging.

[35] Liew A. W. C., Yan H. (2003). An adaptive spatial fuzzy clustering algorithm for 3-D MR image segmentation. *IEEE Transactions on Medical Imaging*.

[36] Li C., Huang R., Ding Z., Gatenby C., Metaxas D., Gore J. A variational level set approach to segmentation and bias correction of images with intensity inhomogeneity.

[37] Milles J., Zhu Y. M., Gimenez G., Guttmann C. R., Magnin I. E. (2007). MRI intensity nonuniformity correction using simultaneously spatial and gray-level histogram information. *Computerized Medical Imaging and Graphics*.

[38] Xian M., Zhang Y., Cheng H. D. (2018). A benchmark for breast ultrasound image segmentation (BUSIS). http://arxiv.org/abs/1801.03182.

[39] Lee C. Y., Chou Y. H., Huang C. S., Chang Y. C., Tiu C. M., Chen C. M. (2010). Intensity inhomogeneity correction for the breast sonogram: constrained fuzzy cell-based bipartitioning and polynomial surface modeling. *Medical Physics*.

[40] Szegedy C., Vanhoucke V., Ioffe S., Shlens J., Wojna Z. Rethinking the inception architecture for computer vision.

[41] He K., Zhang X., Ren S., Sun J. Deep residual learning for image recognition.

[42] Huang G., Liu Z., Van Der Maaten L., Weinberger K. Q. Densely connected convolutional networks.

[43] Xiao T., Liu L., Li K., Qin W., Yu S., Li Z. (2018). Comparison of transferred deep neural networks in ultrasonic breast masses discrimination. *BioMed Research International*.

[44] Han S., Kang H. K., Jeong J. Y. (2017). A deep learning framework for supporting the classification of breast lesions in ultrasound images. *Physics in Medicine & Biology*.

